# Being Human: A Qualitative Interview Study Exploring Why a Telehealth Intervention for Management of Chronic Conditions Had a Modest Effect

**DOI:** 10.2196/jmir.5879

**Published:** 2016-06-30

**Authors:** Alicia O'Cathain, Sarah J Drabble, Alexis Foster, Kimberley Horspool, Louisa Edwards, Clare Thomas, Chris Salisbury

**Affiliations:** ^1^ Medical Care Research Unit School of Health and Related Research University of Sheffield Sheffield United Kingdom; ^2^ Centre for Academic Primary Care School of Social and Community Medicine University of Bristol Bristol United Kingdom

**Keywords:** telehealth, depression, cardiovascular diseases, qualitative research, chronic disease, randomized controlled trials, primary health care

## Abstract

**Background:**

Evidence of benefit for telehealth for chronic conditions is mixed. Two linked randomized controlled trials tested the Healthlines Service for 2 chronic conditions: depression and high risk of cardiovascular disease (CVD). This new telehealth service consisted of regular telephone calls from nonclinical, trained health advisers who followed standardized scripts generated by interactive software. Advisors facilitated self-management by supporting participants to use Web-based resources and helped to optimize medication, improve treatment adherence, and encourage healthier lifestyles. Participants were recruited from primary care. The trials identified moderate (for depression) or partial (for CVD risk) effectiveness of the Healthlines Service.

**Objective:**

An embedded qualitative study was undertaken to help explain the results of the 2 trials by exploring mechanisms of action, context, and implementation of the intervention.

**Methods:**

Qualitative interview study of 21 staff providing usual health care or involved in the intervention and 24 patients receiving the intervention.

**Results:**

Interviewees described improved outcomes in some patients, which they attributed to the intervention, describing how components of the model on which the intervention was based helped to achieve benefits. Implementation of the intervention occurred largely as planned. However, contextual issues in patients’ lives and some problems with implementation may have reduced the size of effect of the intervention. For depression, patients’ lives and preferences affected engagement with the intervention: these largely working-age patients had busy and complex lives, which affected their ability to engage, and some patients preferred a therapist-based approach to the cognitive behavioral therapy on offer. For CVD risk, patients’ motivations adversely affected the intervention whereby some patients joined the trial for general health improvement or from altruism, rather than motivation to make lifestyle changes to address their specific risk factors. Implementation was not optimal in the early part of the CVD risk trial owing to technical difficulties and the need to adapt the intervention for use in practice. For both conditions, enthusiastic and motivated staff offering continuity of intervention delivery tailored to individual patients’ needs were identified as important for patient engagement with telehealth; this was not delivered consistently, particularly in the early stages of the trials. Finally, there was a lack of active engagement from primary care.

**Conclusions:**

The conceptual model was supported and could be used to develop further telehealth interventions for chronic conditions. It may be possible to increase the effectiveness of this, and similar interventions, by attending to the human as well as the technical aspects of telehealth: offering it to patients actively wanting the intervention, ensuring continuity of delivery by enthusiastic and motivated staff, and encouraging active engagement from primary care staff.

## Introduction

The increasing prevalence of chronic conditions presents a challenge to health systems internationally in terms of the ability to meet patients’ health care needs. There is interest in the potential of technology to address this challenge by offering an alternative to face-to-face care between health care professionals and patients [1]. Telemedicine or telehealth delivers health care at a distance using information and communication technologies for diagnosis, treatment, and prevention of health problems [1]. These technologies can be supported by different types of clinical and nonclinical staff and thus expand health care provision and increase access to care. Policy makers worldwide have enrolled large numbers of patients in telehealth schemes [1-3] and are evaluating telehealth programs [1,4].

Despite the promotion of telehealth internationally, evidence of benefit is mixed [5-7]. A large review of the effectiveness of telehealth for chronic conditions concluded that the evidence base is weak and inconclusive due to publication bias, short-term outcome measurement, and a lack of focus on cost-effectiveness [5]. A review of reviews of telehealth concluded that telehealth could be effective for the management of some chronic conditions, but that evidence is mixed with a need for larger studies [6]. A more recent review of interactive telehealth concluded that telehealth was effective for some chronic conditions, specifically heart failure and diabetes, but that evidence was inconsistent for other conditions [7].

The lack of consistency of the evidence base on telehealth could reflect a lack of theoretical underpinning for many interventions or problems with the quality of their evaluation. It has been recommended that large, rigorous evaluations of any new interventions are undertaken [8]. Furthermore, process evaluations undertaken alongside trials of complex interventions such as telehealth may enable researchers to understand why interventions succeed or fail by exploring context, mechanisms of action, and implementation of the intervention [9]. Qualitative research can contribute to this [10].

Researchers have started to address the need for large, pragmatic trials of theory-based telehealth for chronic conditions. Two large, linked, randomized controlled trials (RCTs) of a telehealth intervention, known as the Healthlines Service, which followed up patients for a year, focused on depression and on risk factors for cardiovascular disease (CVD) [11]. The trial targeting depression identified a moderate clinical benefit [12], whereas the trial focusing on reducing risk factors for CVD identified a partial effect; that is, improvement in some individual risk factors but not overall CVD risk score [13]. An embedded qualitative study was undertaken with both these trials with the aim of explaining the results of the trials [11]. In this paper, we report this embedded qualitative study to explore why the trials showed modest effects only and then discuss the implications of this for future use and evaluation of this type of telehealth intervention.

## Methods

The telehealth intervention is described in Textbox 1. The 2 RCTs are described in Textbox 2.

**The intervention**

The content and delivery of the Healthlines Service was underpinned by a conceptual model called the TElehealth in CHronic disease (TECH) model [14]. This model was constructed by the research team based on an extensive review of quantitative and qualitative evidence, a qualitative interview study with patients and staff experienced with telehealth or with chronic conditions [15] and a postal survey of patients’ levels of interest in different forms of telehealth [16]. The TECH model builds on the Chronic Care Model [17] and proposes that telehealth interventions are most likely to be effective and acceptable if they address (1) engagement of patients and health professionals; (2) effective chronic disease management (including self-management, optimization of treatment, care coordination); (3) partnership between providers; and (4) patient and health system context. This model was used to design a telehealth intervention for 2 exemplar chronic conditions: depression and raised CVD risk.

The Healthlines Service was based on regular telephone calls over a 12-month period from a nonclinical health information advisor (HIA). The content of the calls was guided by scripts within computer software designed for the study. The HIAs also used motivational interviewing skills to encourage behavior change and improve self-management. Patients were encouraged to identify goals and then offered links to information about quality-assessed resources on the Internet. Some aspects of the intervention were condition specific. For patients with depression, the intervention included book-based or computerized cognitive behavioral therapy and access to a Web-based mental health network (Big White Wall). For patients with raised CVD risk, the intervention included blood pressure self-monitoring using loaned blood pressure monitors with automated feedback via a Web portal and advice about diet, exercise, and smoking cessation. For both conditions, patients’ use of medication was reviewed by the HIA. Problems with medication adherence were addressed, where patients were not being treated in accordance with national guidelines; a treatment recommendation was sent to their general practitioner and copied to the Web portal where the patient could view it. The intervention was designed to complement primary care delivered in general practice. The intervention was delivered by NHS Direct, which was a national telephone-based service at the time of the study. The staff members delivering the intervention were experienced HIAs who were given additional training to deliver the Healthlines Service.

**The trials**

The Healthlines Service was tested in 2 linked, pragmatic randomized controlled trials comparing the intervention plus usual care versus usual care alone. Usual care for depression was attendance at general practice, including use of medication and possible referral to psychological services. Usual care for raised CVD risk was attendance at general practice where patients might receive blood pressure monitoring, medication, and lifestyle advice. The trials were undertaken with adults with depression or raised CVD risk recruited from 43 general practices in 3 areas of England. Both trials were powered to detect odds ratios of 1.7 with 80% power [11]. In total, 609 patients were recruited to the depression trial. The primary outcome was response to treatment measured using the Patient Health Questionnaire (PHQ-9) [18] and defined as a reduction ≥5 points and score <10 after 4 months. The treatment response was higher in the intervention group than in the control group (27% (68/255) vs 19% (50/270), odds ratio=1.7 (95% CI 1.1-2.5; *P*=.02). Twenty-five percent (78/307) of patients received little or none of the intervention [12], which is a similar rate to other pragmatic trials of telehealth for depression.

Overall, 641 people were recruited to the CVD risk trial. The primary outcome was response to treatment defined as maintenance or reduction in 10-year risk of CVD (measured by QRISK2 score [19]) after 12 months. Participants receiving the intervention had a modest response to treatment compared with those receiving usual care (50% (148/295) vs 43% (124/291), respectively; adjusted odds ratio 1.3; 95% CI 1.0-1.9). The intervention was associated with reductions in blood pressure (difference in mean systolic -2.7 mm Hg (95% CI -4.7 to -0.6)) and weight (-1.0 kg (95% CI -1.8 to -0.3)) but not in cholesterol or smoking status. Eight percent (26/325) of intervention participants received little or none of the intervention, and a third (103/325) received the full course of intended telephone encounters over the course of a year [13].

We undertook a qualitative interview study alongside the 2 trials to explain the results of the trials. We planned to interview 3 groups who could reflect on the intervention: primary care staff working in collaboration with the intervention who could offer perspectives on its feasibility and acceptability to primary care; staff from the organization delivering the intervention (NHS Direct) who could offer perspectives on feasibility; and patients who had experienced the intervention who could reflect on its acceptability. We chose to use the data collection method of interviews because they allow in-depth exploration of individuals’ perceptions.

### Sampling

For the first group (primary care staff), we planned to sample 6 general practices, selected to include practices with populations from varying levels of deprivation. We had to widen our original sample from 6 to 13 general practices because it proved difficult to recruit sufficient numbers of primary care staff from the original set of practices. Within the 13 practices, we sampled purposively to reflect the range of relevant professionals offering primary health care to participants within the intervention arm of the trial: general practitioners (GPs) and practice nurses or health care assistants.

For the second group (NHS Direct staff), we sampled staff purposively to include those delivering the intervention to participants (Health Information Advisors—HIAs), those offering technical expertise for the intervention, and those involved in team and strategic management.

For the third group (patients), we first sampled patients purposively from the intervention arm of the trials to ensure half of interviewees were in the depression trial and half in the CVD risk trial. Next, we used maximum variation sampling so that patients were interviewed who differed in terms of gender, age, and levels of depression or types of CVD risk factors. As a large proportion of patients using the intervention for depression used little or none of the intervention (25% in depression trial vs 8% in CVD risk trial), we also interviewed some patients who had withdrawn from the depression intervention.

### Data Collection

For primary care staff, we wrote to GPs and practice nurses in participating practices asking for consent for an interview. We interviewed primary care staff at different stages of the trial period to obtain a mix of views at an early and later stage of the intervention delivery. Interviews took place face to face at their workplace or by telephone if this was more convenient.

For NHS Direct staff, we approached senior managers to identify relevant staff. We interviewed staff in July 2013, around 12 months after the first depression participant was randomized and 8 months after the first CVD risk participant was randomized. This allowed staff to reflect on both the early and later stages of intervention delivery.

For patients, we contacted those recruited to the intervention arm of the trials who had consented to participate in the interview study during the trial recruitment process. We interviewed these patients after at least 4 months (depression) or 6 months (CVD risk) of experiencing the intervention to allow us to obtain reflections on different stages of their care. This was after the primary outcome measure had been collected in the depression trial (4 months) and after the first collection of follow-up outcome data (at 6 months) in the CVD risk trial. Patients who had withdrawn from the intervention were interviewed within 5 months of recruitment. Face-to-face interviews with patients took place at their home or an alternative venue, depending on their preference.

SJD undertook most of the interviews, with support from AF and KH. We obtained written informed consent from all interviewees. Regardless of interviewee type, the focus of the interviews was on the intervention. We asked about its perceived utility, problems arising, and issues that enhanced or hindered its operation in practice. In addition, we asked about the components of the conceptual model underlying the intervention: engagement, promoting self-management, treatment optimization, care coordination, partnership, and context. Interviews lasted on average 45 minutes for staff, ranging from 16 to 88 minutes, and 58 minutes for patients, ranging from 21 to 124 minutes.

### Analysis

Interviews were digitally recorded and transcribed verbatim. The framework approach was used to analyze the data [20]. We read some transcripts from each type of interviewee for familiarization (stage 1 of framework analysis). We constructed a thematic framework based on reading these transcripts and the functions of context, mechanisms of action, and implementation important to process evaluations [9] (stage 2 of framework analysis). As this qualitative study was embedded within RCTs, we supplemented this approach with a framework of the use of qualitative research with trials [10]. This permitted further exploration of themes concerning the trial, outcomes, and the health conditions under study. Subthemes of the theme “mechanisms of action” were informed by the components of the TElehealth in CHronic disease (TECH) model: engagement, promoting self-management, treatment optimization, care coordination, partnership, and context [14].

SJD coded all transcripts to the thematic framework, adding emerging subthemes throughout this process (stage 3 of framework analysis). SJD, AOC, AF, and KH then read the text within each subtheme, paying attention to which interviewees contributed to each subtheme. The final stage of the framework approach—“mapping and interpretation”—involved consideration of relationships between themes and subthemes. As recommended, the analysis was undertaken before any team member knowing the outcomes of the trials [21]. Findings of the qualitative study were discussed among the research team in September 2014 before the trial results were known. We suggested in our conclusions from this analysis that the intervention would be effective because there was evidence within our data that components of the conceptual model helped some patients in both trials, and the intervention was implemented largely as planned. We also suggested that the complexity of patients’ lives and how the intervention was implemented appeared to diminish its impact. Paying attention to the balance of issues, we predicted a small-to-moderate benefit for each trial. In a second stage of analysis, after the trial outcomes were known in December 2014, we used the findings of this qualitative work to help explain the results of the trials. This involved focusing on the themes we considered to be most relevant to the research question of why this intervention had produced a modest effect, while taking care to acknowledge the uncertainties around our explanation.

The trials and qualitative study were approved by the National Research Ethics Service Committee South West—Frenchay (reference 12/SW/0009) and had the following trial registrations: ISRCTN14172341 (depression) and ISRCTN27508731 (CVD risk).

## Results

### Description of Participants

We undertook 45 interviews in total, with 21 staff and 24 patients.

#### Staff

We interviewed 6 GPs, 5 practice nurses, 1 health care assistant, and 1 practice-based research nurse (13 in total) from 13 of the general practices that had participated in the trials. We approached practice staff who had been involved to some extent in the trials, for example, GPs who had screened lists of potential trial participants before recruitment. From a total of 24 primary care staff approached for interview, 7 GPs and 4 practice nurses declined primarily because they did not feel they had anything to say about the intervention.

We interviewed 8 staff from NHS Direct. This included 4 HIAs who had delivered the intervention for varying lengths of time. Two had worked in the Healthlines Service from the beginning, 1 for a few months, and 1 had been in post for a month at the time of the interview. We also interviewed a strategic manager who had been involved in leading the intervention development, a technical manager who had helped to develop the intervention, a supervisor of the HIAs and a team manager from the wider organization who was not directly involved with the intervention but who managed the HIAs as part of larger team. This latter interview was undertaken to explore the wider organizational context in which the intervention was delivered.

#### Patients

We approached 16 depression and 20 CVD risk trial participants to obtain 12 interviews with each group. Patients declined to participate because they said they were not interested (n=6), were too busy (n=3), could not be contacted (n=1), or did not attend the arranged interview (n=2).

Interviewees participating in the depression trial were interviewed a median of 8 months after randomization, varying between 5 and 10 months. There were 7 women, they were all white, and mainly middle-aged (age range 30-66 years). This generally reflected the demographics of participants in the depression trial. According to the baseline PHQ-9 classifications, 1 interviewee had severe depression, 4 interviewees had moderately severe depression, and the remainder had moderate depression. Four interviewees had formally withdrawn from using the intervention at the time of the interview.

CVD risk interviewees were interviewed a median of 8 months after randomization, varying between 3 and 11 months. They were mainly men (n=9), all white, and all older (age range 62-75 years). This demographic mix was largely in line with the participants in the CVD risk trial. They had a mix of CVD risk factors at baseline: 2 smoked, 9 had a body mass index (BMI) ≥ 30, and 8 had systolic blood pressure ≥ 140. Eight were on blood pressure–lowering medication. The CVD risk score (QRISK2) was high for all interviewees (as that was an inclusion criteria for the trial), ranging between 21% and 58%; 3 had a score higher than 45%. None of the interviewees had formally withdrawn from using the intervention at the time of the interview.

### Overview of Findings

The findings are presented using the framework of mechanisms of action, context, and implementation. We show that interviewees perceived that the intervention was useful for some patients and described aspects of the intervention that they valued. However, contextual issues and problems with implementation negatively affected the impact of the intervention. Quotes are accompanied by labels showing the type of staff or characteristics of patients.

#### Mechanisms of Action

##### Perceptions That the Intervention Was Useful for Some Patients

Interviewees perceived that the intervention had improved the health of some patients. First, staff delivering the intervention described individual patients reporting improved mood and weight loss. They did not describe the characteristics of these patients, but, instead, described the characteristics of patients who they perceived were not being helped by the intervention (see the following section). Second, some of the patients interviewed reported improvements in health, which they associated with the intervention. Among the patients in the depression trial, 6 described benefits such as feeling more positive because they had been shown ways to cope, had learned to share problems with their family, felt listened to, or felt that someone cared about them:

what I needed was a way of dealing with the great sadnesses and a way of coming to terms with it, and I think I have got that from [pause], from The Healthlines Study”

Dep 8, female, aged 66, with moderate depression at baseline

Nine of the twelve CVD risk interviewees had a BMI of 30 or over at baseline. Three of these reported weight loss, which they attributed to the intervention. They were delighted with the amount of weight they had lost since joining the study and described other positive consequences, including reduced blood pressure, ability to walk more easily, and having more energy. Some CVD risk interviewees reported making lifestyle changes that could affect CVD risk factors, such as exercising more, eating more healthily, and reducing alcohol intake. Four of the eight interviewees with high blood pressure at baseline (systolic above 140 mm Hg) reported lowered blood pressure, and another reported reduced use of blood pressure medication related to the intervention. Improvements in blood pressure were attributed to weight loss or introduction of blood pressure medication:

*Interviewer: You have got high blood pressure I am presuming?*

CVD participant: Not anymore.

*Interviewer: Not anymore, good (laugh)*

CVD participant: Mainly thanks to this system

CVD risk 8, male, aged 70, with high blood pressure at baseline

##### Aspects of the Intervention Valued by Staff and Patients

When asked about the different components of the intervention, interviewees tended to describe their value and how they helped to improve health. That is, there was support for the conceptual model on which the intervention was based. For example, both the HIAs delivering the intervention and patients receiving it described the necessity and value of different aspects of the intervention aimed at encouraging patient engagement. This included the technical support for patients, which helped them to use computer-based aspects of the intervention, the continuity of contact with the same HIA, which helped to build rapport with patients, and enthusiastic and motivated HIAs who made the effort to tailor the intervention to patients’ needs:

it has been good to build up some kind of relationship

CVD risk 11, female, aged 49 years, overweight at baseline

There was also support for the value of the self-management aspect of the intervention. Most of the patients we interviewed described how the intervention helped them to develop self-management skills through raising awareness of their health problems and educating them about ways of dealing with those problems. As 1 patient put it, the intervention was about

helping myself to help myself

Dep 2, male, aged 60 years, with moderately severe depression at baseline.

I think it makes people realize that there are things that you can do on a day to day basis […] to bring [their blood pressure] down, if they are checking it that regularly for a purpose. You know, I went out for a walk this morning and my blood pressure was really good today, and things like that. It makes it very obvious in black and white right in front of them that the days when they are doing things, and being a bit more well-behaved if you like, that it does make a difference.

Practice Nurse 113

and then it just gives them something to work on and I make it clear to them all that they have to do the hard work themselves if they want to reach their target. And 8 times out of 10 next time I speak to them they have done it or the first thing they say to me is “well I have been eating off a smaller plate” and it is really nice to hear that.

Health Information Advisor 2

Finally, there was some evidence of medication optimization occurring. Some patients receiving the depression intervention were on antidepressants, and some CVD risk participants were on blood pressure medication and statins. Interviewees reported that the intervention impacted medication taking through HIAs prompting patients to discuss medication with their GP or through letters directly from HIA s to GPs.

#### Context

##### Individual Context: Lack of Fit With Perceived Need

A key contextual issue, which may have impacted the effectiveness of the intervention, was patients’ desire to improve their health. Patients with depression and primary care staff reported long waits for access to usual care services such as counseling and cognitive behavioral therapy. Patients with CVD risk factors wanted to improve their health, and some of those who wanted to improve their lifestyle perceived a lack of advice about how to do this, although the practice nurses we interviewed said they offered this service.

However, there were indications that some patients did not understand what the intervention entailed when they signed up to join the trial and, in fact, had no interest in what was on offer once they had started the intervention. Some patients in the depression trial described the intervention as too superficial, not giving access to a therapist, or the same as previous treatments because they had already tried cognitive behavioral therapy.

Some patients in the CVD risk trial reported low motivation to change their lifestyle; they had been interested generally in improving their health without necessarily understanding that this would entail them making lifestyle changes or had joined the study for altruistic reasons in terms of helping others through participating in research:

and, I thought, well it will help me, but it might help somebody else, that is the reason I had a go

CVD risk 7, male, aged 74 years, with high blood pressure at baseline

Other patients in the CVD risk trial had no intention of addressing a key CVD risk factor that led to their eligibility for the trial. In particular, 2 of our CVD risk interviewees were smokers at baseline. Both these reported no success with smoking cessation because they did not want to stop smoking:

do not bother, I smoke

CVD risk 1, male, aged 62 years, smoker with high blood pressure at baseline.

Health Information Advisors noted that few patients had reported giving up or cutting down smoking and that this was a difficult lifestyle issue to have an impact on. The staff we interviewed believed that if intrinsic motivation to change was absent, then patients, particularly those in the CVD risk trial, would find it difficult to make the necessary lifestyle changes in the timeframe in which the intervention was offered.

##### Individual Context: Lack of Fit With Patients’ Lives

Patients in the depression trial tended to be of working age, whereas those in the CVD risk trial tended to be retired. These younger patients with depression were described by HIAs as too busy due to childcare and employment to engage with key aspects of the intervention such as the telephone calls and homework for the cognitive behavioral therapy. The HIAs wondered whether lack of engagement was due to their depression and their busy lives. They felt that those who did complete the cognitive behavioral therapy course obtained benefit from it, and so they wanted the inclusion criteria for the trial to focus on those who were really committed to making changes and engaging with the intervention:

The depression ones, a large, it seems to be a lot, to me, younger people, a lot more women, not all but they are rushing around, they do not have time, they forget they have got appointments, and whether it is part of depression or not I do not know, but they do not often, they do not answer the phone

Health Information Advisor 1

Some interviewees from the depression trial described serious ongoing life events such as the threat of losing disability and unemployment benefits, physical illnesses, or coping with family members and friends who were very ill or depressed. These issues caused stress on top of the depression, making engagement with the intervention difficult. According to the HIAs in our study, life events preventing engagement with the intervention appeared to be less of an issue for CVD risk patients. Our interviewees with CVD risk factors did not offer the same description of complex lives as our interviewees with depression. The level of complexity of patients’ lives may have been related to age because the patients in the CVD risk trial were older and many were retired. Only 1 of the CVD risk interviewees was still in full-time paid employment, and this interviewee did report finding it difficult to fit the intervention into their life.

##### Research Context: A Randomized Controlled Trial

The intervention was offered in the context of an RCT. The intervention for depression was ready for use at the beginning of the trial and needed little or no adaptation during the trial. However, interviewees from NHS Direct discussed delays in starting the CVD risk intervention at the beginning of the trial due to a number of technical problems with the intervention. This resulted in some patients waiting for several months between randomization and receiving the intervention. As specified in the trial protocol, the primary outcome of the CVD risk trial was measured 12 months after randomization. This resulted in measurement of 12-month outcomes before some patients had completed the intervention, which may have reduced the measured effect of the intervention for CVD risk.

#### Implementation of the Intervention

When we asked the 3 groups of interviewees about different components of the intervention, they not only described the value of these components (see previous sections) but also described how they occurred in practice. With the exception of 3 issues (described in the following sections), their descriptions aligned with the planned implementation of the Healthlines Service.

##### Continuity of Enthusiastic and Motivated Health Information Advisors

Continuity of care—ensuring the same HIAs talked to the same patient throughout their care—was one of the ways in which the intervention delivered the patient engagement component of the TECH conceptual model. This appeared to be very important to some patients we interviewed and was compromised in the early months of implementation. Interviewees from NHS Direct described how, during the earlier months of the intervention, they tested out a model of using staff part-time in the Healthlines Service and part-time in the wider organization. This made it difficult for the same HIA to contact the same patients and also caused challenges for HIAs trying to learn to use a technically complex intervention. It was also compounded by large numbers of patients entering the CVD risk at the same time. This lack of continuity compromised the ability of HIAs to actively tailor the intervention to different patients. As the intervention progressed, NHS Direct changed the model of provision to a small dedicated team of staff who were enthusiastic about the intervention and felt motivated to help patients to improve their health. The HIAs we interviewed were part of this dedicated team and described how they placed emphasis on providing continuity of care and tailoring the intervention to the needs of individual patients. However, they also described how continuity of care could not be fully delivered even in the later stages because the small team sometimes struggled to cover sickness absence and holidays while still providing appointments that suited patients.

The variation in implementation was evident in patients’ descriptions of their experiences. Some of the patients we interviewed appreciated the relationship they had built up with an HIA, feeling listened to and cared for. Others described HIAs as “going through the motions,” rather than attempting to tailor the intervention:

because the spiel was exactly the same

CVD risk 10, male, aged 71 years, with high blood pressure at baseline.

This latter group struggled to engage with the intervention. Indeed, 3 of the interviewees in the depression trial who expressed concern about a protocoled approach had withdrawn from the intervention.

##### Modification of Intervention Delivery During the Trial

NHS Direct staff described how continuing technical difficulties had to be sorted out during the early weeks of using the intervention for CVD risk. Health Information Advisors explained how they had to learn to make the software work in the context of an ongoing conversation with a patient, modifying the flow of the scripts that were built into the intervention to reduce repetition for patients. They also described how they made notes about patients outside the computerized system to help them set and monitor plans for patients.

##### Collaboration With Primary Care

The primary care staff we interviewed had little to say about specific aspects of the intervention. Health Information Advisors and patients described how GPs responded to prompts to consult with patients or change medication but also described how they did not take an interest in patients’ experiences of the intervention or proactively contact the Healthlines Service about individual patients. There was also some evidence that communication between primary care and HIAs did not always reach the level of partnership intended by the conceptual model, which could cause confusion for some patients. For example, GPs did not necessarily agree with advice from the intervention, which was based on national guidelines:

there was this one particular patient who was constantly being, it was being suggested that he be reviewed by the GP. And the GP was reviewing him, but it was still the same, you know, it was a bit, you know, flogging a bit of a dead horse really, because she was, the GP was very happy with the blood pressure. Healthlines Study staff were saying, oh, no, no, no you need to go and see the GP […] and of course the patient is the one caught in the middle

Practice Nurse 111

## Discussion

### Principal Findings

The interviewees described improved outcomes in some patients receiving the intervention. They attributed these improvements to the intervention, describing how components of the conceptual model on which the intervention was based helped to achieve benefit. Aspects of the intervention addressing patient engagement, self-management, and medication optimization were valued. Implementation of the intervention occurred largely as planned. However, problems related to context and implementation may have reduced the size of effect. For depression, the context of patients’ lives was often complex, resulting in these working-age patients sometimes being unable to engage with the intervention. Some patients also wanted a more therapist-based approach rather than the cognitive behavioral therapy on offer. For CVD risk, contextual issues included some patients joining the trial in the hope of improving their health generally, or altruistically helping with research, rather than being motivated to make lifestyle changes to address their specific risk factors. In addition, implementation was not optimal in the early part of the CVD risk trial as technical difficulties with the intervention were addressed and staff delivering the intervention adapted it for use in practice. For both conditions, enthusiastic and motivated staff members offering continuity of intervention delivery tailored to individual patients’ needs were identified as important for patient engagement with telehealth, but this was not delivered consistently, particularly in the early stages of the trials. Finally, there was a lack of active engagement with the intervention from GPs in primary care. Although some of these issues related to the technological aspects of the intervention, most of them are related to human issues—the complexity of patients’ lives and the need for skillful human support to complement the technology.

### Strengths and Limitations

One key strength of this qualitative study was the inclusion of interviews with a wide range of stakeholders: staff offering primary care to patients, managers, and frontline staff delivering the intervention and patients who had used the intervention and those who had withdrawn from it. This greatly improved our understanding of the trial results and provided support for the use of the TECH conceptual model to underpin these kinds of interventions. There were 4 limitations. First, we could have used nonparticipant observation in combination with the interviews, such as listening to telephone calls and observing HIAs in their daily work, which may have helped to further understand implementation of the intervention. Second, although we felt that we achieved data saturation at the data collection stage for most of the groups we targeted, this was not the case for participants in the CVD risk trial because of the range of risk factors they had. Third, inclusion of other groups may have helped to further understand the intervention; in particular, HIAs who had left the service and patients in the control arm of the trials. Finally, we completed our data collection before the end of the intervention. The organizational context in which the intervention was delivered changed toward the end of intervention delivery. NHS Direct ceased to operate toward the end of the trials, although the intervention continued to be offered by the same HIAs working for a different organization. During the change in the organization hosting the service, there was a pause in service delivery for some patients, and this might have affected their engagement with the intervention. However, we did not have data from those delivering or receiving the intervention during or after this change.

### Comparison With Prior Work

Some patients in the trials did not engage with the intervention: 25% of patients in the depression trial and 8% of patients in the CVD risk trial used little or none of the intervention [12,13]. These rates were smaller than a trial of a Web-based program for reducing CVD risk where almost half of the intervention users had dropped out at 12 months [22]. Interestingly, the qualitative research undertaken alongside that trial recommended the addition of human interaction to motivate and engage patients. Our qualitative study identified that motivated staff could enhance patient engagement, and that engagement was also dependent on human factors related to the patients. It identified that patients wanted help with their health, but not necessarily the intervention on offer, or did not see the intervention as a priority in their complex or busy lives. This finding is similar to a systematic review of computerized cognitive behavioral therapy—a core component of our intervention for depression—which identified that a median of 56% participants completed a full course and that personal circumstance was more commonly cited as the cause for noncompletion than difficulties with the technology or social background [23]. We know that a large proportion (82%) of invited patients chose not to participate in our trials in the first place [24]. Among those actively declining participation (rather than not replying to the trial invite), common reasons given were that they were too busy or they were not interested in the research. It was also the case that some patients agreed to participate who did not want the intervention on offer or whose lives were too complex to make use of it. Although efforts were made to communicate to potential participants in advance about what the intervention entailed, it is possible that the nature of the intervention was not described clearly enough and was misunderstood, or that participants held expectations of the intervention that differed from their experience. These patients might have declined to take part in the trial if they had known more about the content of the intervention and the efforts required of them.

Researchers are beginning to test ways of increasing the acceptance of Internet-based mental health interventions using an informational video [25]. This type of video may also be useful when recruiting patients for trials of telehealth interventions to help them make informed choices about participation. For example, people who smoke and do not want to stop might decline to participate if they understand that a key focus on the intervention is to help them reduce this risk factor. This may reflect the real world more because, in practice, patients tend to access smoking cessation services if motivated to stop smoking. It is possible that a future trial with more emphasis on communicating the content of the intervention, and the efforts required by patients to obtain benefit, might result in larger effect sizes than seen here.

The importance of the human aspect of telehealth, in terms of who delivers the intervention and how, was evident from these interviews. This “personal context” of factors, related to the practitioners involved, in terms of their perceptions of the relevance of and interest in the intervention, their skills, and their motivation has been identified as a type of context affecting how interventions work [26]. In our study, this personal context of motivated HIAs appeared to facilitate patient engagement with the intervention through both developing rapport and tailoring the intervention. These 2 issues have been identified as mechanisms of action of telehealth for chronic conditions [27]. Other researchers have also identified the importance of continuity of the person delivering telehealth and their level of motivation during delivery [23,28-30]. This has also been identified as important for the self-management of chronic conditions more generally. For example, a recent systematic review of interventions for the self-management of asthma identified the importance of actively engaging patients and having motivated professionals delivering interventions [31]. This focus on the importance of motivated humans delivering telehealth has not been identified consistently. For example, qualitative research alongside an RCT of an educational Web-based tool to prevent problems in young people whose parents had mental health problems identified technical problems as the key barrier [32].

The lack of proactive engagement with the intervention from primary care was perhaps not too surprising given earlier interviews with practice nurses and GPs before developing the intervention [15]. These health professionals were ambivalent and often skeptical about the contribution of telehealth to the care of chronic conditions. The conclusion of this earlier research was that there was work to be done in terms of helping primary care health professionals to understand the changes in roles and new ways of working necessary to facilitate the introduction and integration of telehealth innovations into their services. Our conclusions after delivery of the intervention were similar, in that there is a need to develop better strategies for primary care engagement with telehealth. This lack of primary care engagement with interventions aimed at chronic conditions is not specific to telehealth interventions [33].

### Implications

When delivering this or similar interventions in the real world, service providers may wish to consider communicating the content of the intervention clearly to prospective users and the amount of time and effort required by them to obtain benefit. They may also wish to ensure the service is provided by motivated staff who can offer continuity of care and tailor the intervention to patients’ needs. Given the lack of engagement from primary care, it may also be helpful for future interventions to try to develop better strategies for primary care engagement that also take into consideration the heavy workload in general practice in the United Kingdom currently. These actions may increase the effect of this or similar interventions in the future. There are also implications for the treatment of other chronic conditions. The conceptual model for the intervention was supported by this qualitative research, and so could be used to develop further interventions tailored for different conditions. These interventions would have to undergo rigorous evaluation in RCTs. Finally, there is a methodological implication for trialists. Due to technical problems and delays, some aspects of the intervention were not fully functional during the early months of the trials, particularly for CVD risk. The possible implications of this are that participants in the early stage of the trials may have received an underdeveloped intervention. Feasibility testing before a full evaluation is an important aspect of the evaluation of complex interventions [34], although finding sufficient resources to do so within a fixed research timeline and budget when the problems cannot necessarily be anticipated in advance may be challenging.

### Conclusions

This qualitative research helped to explain why the outcomes of 2 linked trials were modest. The conceptual model of the intervention was supported and could be used to develop further telehealth interventions for chronic conditions. It may be possible to increase the effectiveness of this, and similar interventions, by attending to the human and the technical aspects of telehealth: offering it to patients actively wanting the intervention, ensuring continuity of delivery by enthusiastic and motivated staff, and encouraging active engagement from primary care.

## Abbreviations

BMI: body mass index
CVD: cardiovascular disease
GP: general practitioner
HIA: health information advisor
PHQ-9: Patient Health Questionnaire
RCT: randomized controlled trial
TECH: TElehealth in CHronic disease
